# Precision Nursing: advances and challenges in implementation*

**DOI:** 10.1590/1518-8345.8046.4683

**Published:** 2025-10-27

**Authors:** Luís Carlos Lopes-Júnior

**Affiliations:** 1 Universidade Federal do Espírito Santo, Vitória, ES, Brazil; 2 Scholarship holder at the Conselho Nacional de Desenvolvimento Científico e Tecnológico (CNPq), Brazil

## Abstract

**(1)** Precision Nursing (PN) emerges as an innovative field within Nursing. **(2)** PN represents the shift from standardized care to a highly tailored approach. **(3)** Nursing is strategically positioned to lead the translation of these advancements into practice. **(4)** PN reinforces a more tailored, predictive, and participatory approach to nursing care. **(5)** PN has the potential to become a cornerstone in the transformation of Modern Nursing.

Precision Nursing (PN) emerges as an innovative field within Nursing, driven by advancements in Precision Medicine (PM) and the “omics” sciences^(1-2)^. PN represents a shift from standardized care to a highly tailored approach based on clinical, physiological, environmental, social, and behavioral data^(3)^. This shift emphasizes not only the care process but also health promotion, disease prevention, and early intervention. Additionally, PN emphasizes individualized care, expecting nurses to deliver precise and competent care to patients with varied needs^(2-3)^. Thus, PN is grounded in each individual’s uniqueness, taking into account their health history, personal preferences, and the best available scientific evidence to provide personalized care across the entire lifespan^(1-3)^. The transition from structural genomics to functional genomics has enabled the integration of various omics sciences — such as proteomics, epigenomics, transcriptomics, pharmacogenomics, and metabolomics — into clinical practice, paving the way for a new era of personalized care^(1,3)^.

Thanks to its close connection with patients and focus on holistic care, Nursing is uniquely positioned to translate these advances into safe and effective clinical interventions. PN not only redefines individualized care but also broadens the scope of evidence-based practice by integrating biomarkers and technological tools for more precise and responsive monitoring^(1-2)^.

Worldwide, nurses have been expanding their scope of practice by identifying biomarkers linked to conditions such as chronic stress in children and adolescents, frailty in older adults, vascular access maintenance, pressure injury prevention, and cognitive impairment^(1-3)^. Other nursing professionals have been utilizing artificial intelligence to predict the risk of falls and pressure injuries, while also investigating biomarkers in non-pharmacological interventions to optimize clinical outcomes^(4-5)^. Moreover, the emphasis on prevention, management, and symptom relief represents one of the key contributions of PN, aiming to reduce symptoms and enhance quality of life^(5)^.

The incorporation of these technologies into everyday Nursing practice enhances nurses’ ability to deliver more predictive, preventive, and participatory care^(1)^. Despite PN’s transformative potential, challenges persist. These challenges encompass the need for specialized training in omics sciences, the integration of data from diverse sources to enable personalized care, and the assurance of equitable access to emerging technologies^(1-2)^. Improving data infrastructure and adopting translational research approaches are essential to successfully implementing PN in clinical practice^(3)^.

Traditionally, clinical data are collected through methods such as standardized questionnaires, health service records, electronic medical records, omics reports, imaging tests, and physiological monitoring. However, emerging technologies—such as mobile health devices (*mHealth*), non-invasive sensors like *actigraphy*, and passive monitoring systems—have been incorporated to provide more detailed analyses of individuals’ biological, behavioral, and environmental characteristics^(1-2)^. This information plays a crucial role in guiding personalized interventions, thereby improving symptom management and self-care for individuals most likely to benefit from therapeutic strategies^(1-2)^.

Addressing both equity and precision health is crucial, given the significant impact of socioeconomic, cultural, and environmental factors on treatment response^(1,6)^. Another key challenge is the integration of technology into the Precision Era, which raises complex ethical questions and has significant implications for global health systems^(1,6)^.

Training and preparing nurses in omics sciences and disruptive technologies critical to personalized care continues to be a significant challenge. To meet this goal, undergraduate and graduate programs must be redesigned to include these subjects and adequately prepare nurses to practice PN^(1,7)^. Continuing education and professional development are essential for empowering nurses to identify application areas, conduct research, and foster innovation in healthcare^(7)^. Oncology stands out as a leading field in Precision Nursing, where nurses directly utilize omics sciences to prevent, diagnose, and treat cancer through genetic testing, biomarkers, pharmacogenomics, proteomics, and bioinformatics^(1,7)^.

A seamless transfer of research knowledge into clinical practice is essential for solidifying PN, as it facilitates decision-making and enables personalized care^(1,7)^. Despite the challenges, translational research in Nursing holds great potential to deliver significant benefits to patients and their families, positively affecting quality of life and clinical outcomes.

PN represents an innovative approach with the potential to transform healthcare. However, implementing it requires developing specialized skills, integrating emerging technologies, and reinforcing policies that guarantee equitable access to innovations. By overcoming these challenges, Nursing can lead the way in providing care that is increasingly personalized, effective, and humanized.

Therefore, the concept of Precision Nursing is not distant from nurses’ daily practice. In fact, it represents a practical and essential approach to fostering human-centered care, based on strong evidence and the incorporation of new technologies. By overcoming challenges such as workforce training and data infrastructure, PN can establish itself as a cornerstone in the transformation of modern Nursing.

## Data Availability

All data generated or analysed during this study are included in this published article.

## References

[1] Harrington L. (2021). Precision Nursing. AACN Adv Crit Care.

[2] González Chordá V. M. (2024). Precision nursing and personalized care. Enferm Clin (Engl Ed).

[3] Ielapi N., Andreucci M., Licastro N., Faga T., Grande R., Buffone G. (2020). Precision Medicine and Precision Nursing: The Era of Biomarkers and Precision Health. Int J Gen Med.

[4] Ladios-Martin M., Cabañero-Martínez M. J., Fernández-de-Maya J., Ballesta-López F. J., Belso-Garzas A., Zamora-Aznar F. M. (2022). Development of a predictive inpatient falls risk model using machine learning. J Nurs Manag.

[5] Hickey K. T., Bakken S., Byrne M. W., Bailey D. C. E., Demiris G., Docherty S. L. (2020). Precision health: Advancing symptom and self-management science. Nurs Outlook.

[6] Burke W., Thummel K. (2019). Precision medicine and health disparities: The case of pediatric acute lymphoblastic leukemia. Nurs Outlook.

[7] Lopes-Júnior L. C. (2021). Personalized Nursing Care in Precision-Medicine Era. SAGE Open Nurs.

