# Supplementary material for: Precision Nursing: advances and challenges in implementation
Source: Rev Lat Am Enfermagem. 2025 Oct 27;33:e4683. doi: 10.1590/1518-8345.8046.4683 (PMC12562620; doi:10.1590/1518-8345.8046.4683)
Supplement: Translation

# Translation: Enfermagem de Precisão: avanços e desafios na implementação*

**DOI:** 10.1590/1518-8345.8046.4684

**Authors:** Luís Carlos Lopes-Júnior

**Affiliations:** 1 Universidade Federal do Espírito Santo, Vitória, ES, Brasil; 2 Bolsista do Conselho Nacional de Desenvolvimento Científico e Tecnológico (CNPq), Brasil

## Abstract

**(1)** A Enfermagem de Precisão (EP) emerge como um campo inovador dentro da Enfermagem. **(2)** A EP representa a transição de um atendimento padronizado para uma abordagem altamente personalizada. **(3)** A Enfermagem está estrategicamente posicionada para liderar a translação desse avanço. **(4)** A EP reitera um cuidado de enfermagem mais personalizado, preditivo e participativo. **(5)** A EP pode se consolidar como um pilar na transformação da Enfermagem Moderna.

A Enfermagem de Precisão (EP) emerge como um campo inovador dentro da Enfermagem, impulsionada pelos avanços da Medicina de Precisão (MP) e das ciências “ômicas”^(1-2)^. A EP representa a transição de um atendimento padronizado para uma abordagem altamente personalizada, baseada em dados clínicos, fisiológicos, ambientais, sociais e comportamentais^(3)^. Essa mudança enfatiza não apenas o tratamento, mas também a promoção da saúde, a prevenção de doenças e intervenções precoces. Em adição, a EP centra-se no atendimento individualizado, exigindo que enfermeiros atuem com precisão e competência ao lidar com pacientes que apresentam necessidades diversas^(2-3)^. Dessa forma, a EP fundamenta-se na singularidade de cada indivíduo, levando em consideração o histórico de saúde e as preferências dos pacientes e as melhores evidências científicas disponíveis para oferecer um cuidado personalizado ao longo de todo o ciclo de vida^(1-3)^. A transição da genômica estrutural para a genômica funcional permitiu a integração das diversas ciências ômicas, tais como a proteômica, epigenômica, transcriptômica, farmacogenômica e metabolômica na prática clínica, trazendo uma nova era de personalização do cuidado^(1,3)^.

A Enfermagem, por sua proximidade com o paciente e expertise em cuidado holístico, está estrategicamente posicionada para liderar a translação desses avanços em intervenções clínicas eficazes e seguras. A EP não apenas redefine o cuidado individualizado, mas também amplia o escopo da prática baseada em evidências, incorporando biomarcadores e ferramentas tecnológicas para um acompanhamento mais preciso e responsivo^(1-2)^.

Enfermeiros no mundo todo vêm expandindo sua atuação na identificação de biomarcadores associados a diferentes condições, como estresse crônico em crianças e adolescentes, fragilidade em idosos, manutenção de acesso vascular, prevenção de lesões por pressão e comprometimento cognitivo^(1-3)^. Outros profissionais enfermeiros têm explorado a inteligência artificial para prever o risco de quedas e lesões por pressão, além de investigar biomarcadores em intervenções não farmacológicas para otimizar desfechos clínicos^(4-5)^. Ademais, o foco na prevenção, no manejo e no alívio de sintomas é uma das principais contribuições no campo da EP, voltada para a redução de sintomas e a melhoria da qualidade de vida^(5)^.

A incorporação dessas tecnologias ao cotidiano da Enfermagem reforça a capacidade do enfermeiro em oferecer um cuidado mais preditivo, preventivo e participativo^(1)^. Apesar do potencial transformador da EP, desafios persistem. Entre eles, a necessidade de capacitação específica nas ciências ômicas, a integração de dados de múltiplas fontes para personalização do cuidado e a equidade no acesso às novas tecnologias^(1-2)^. O fortalecimento da infraestrutura de dados e a adoção de modelos de pesquisa translacional são essenciais para garantir a implementação efetiva da EP na prática clínica^(3)^.

Tradicionalmente, os dados clínicos são obtidos por meio de métodos como questionários padronizados, registros de serviços de saúde, prontuários eletrônicos, relatórios ômicos, exames de imagem e monitoramento fisiológico. No entanto, tecnologias emergentes, como dispositivos móveis de saúde (*mHealth*), sensores não invasivos (como *actigraphy*) e sistemas de monitoramento passivo, têm sido incorporadas para fornecer uma análise mais detalhada das características biológicas, comportamentais e ambientais dos indivíduos^(1-2)^. Essas informações são essenciais para a personalização das intervenções, tornando o manejo dos sintomas e do autocuidado mais eficaz para indivíduos com maior propensão a responder positivamente às estratégias terapêuticas^(1-2)^.

A discussão sobre equidade e saúde de precisão também se faz necessária, considerando que fatores socioeconômicos, culturais e ambientais influenciam significativamente a resposta ao tratamento^(1,6)^. Outro grande desafio refere-se à incorporação tecnológica na Era de Precisão, que levanta importantes questões éticas e impactos para os sistemas de saúde globais^(1,6)^.

A preparação e capacitação dos enfermeiros nas ciências ômicas e nas tecnologias disruptivas que sustentam o cuidado personalizado se transpõe como um desafio. Para isso, é essencial reformular os currículos acadêmicos da graduação e pós-graduação, integrando esses conhecimentos e preparando a força de trabalho para atuar na EP^(1,7)^. A formação continuada e o aprimoramento profissional são indispensáveis para que enfermeiros possam identificar áreas de aplicação, conduzir pesquisas e promover inovação em saúde^(7)^. Entre as áreas de maior desenvolvimento da EP, destaca-se a Oncologia, na qual enfermeiros lidam diretamente com as ciências ômicas para prevenção, diagnóstico e tratamento do câncer, utilizando testes genéticos, biomarcadores, farmacogenômica, proteômica e bioinformática^(1,7)^.

Um aspecto fundamental para a consolidação da EP é a transferência do conhecimento gerado por pesquisas para a prática clínica, facilitando a tomada de decisões e provendo o cuidado personalizado^(1,7)^. Apesar dos desafios, a pesquisa translacional em Enfermagem pode gerar benefícios expressivos para pacientes e suas famílias, impactando positivamente a qualidade de vida e os desfechos clínicos.

A EP representa uma abordagem inovadora, com potencial para transformar o cuidado em saúde. Contudo, sua implementação requer o desenvolvimento de competências especializadas, a incorporação de tecnologias emergentes e o fortalecimento de políticas que garantam equidade no acesso às inovações. Ao superar esses desafios, a Enfermagem poderá consolidar-se como protagonista na promoção de um cuidado cada vez mais personalizado, eficiente e humanizado.

Dessa forma, a EP não é um conceito distante da prática diária dos enfermeiros. Pelo contrário, trata-se de uma abordagem viável e necessária para a promoção de um cuidado centrado na pessoa, baseado em evidências robustas e na integração de novas tecnologias. Ao superar desafios como a capacitação e a infraestrutura de dados, a EP pode se consolidar como um pilar fundamental na transformação da Enfermagem moderna.

## Data Availability

Todos os dados gerados ou analisados durante este estudo estão incluídos neste artigo publicado.

# Translation: Enfermería de Precisión: avances y desafíos de la implementación*

**DOI:** 10.1590/1518-8345.8046.4682

**Authors:** Luís Carlos Lopes-Júnior

**Affiliations:** 1 Universidade Federal do Espírito Santo, Vitória, ES, Brasil; 2 Becario del Conselho Nacional de Desenvolvimento Científico e Tecnológico (CNPq), Brasil

## Abstract

**(1)** La Enfermería de Precisión (EP) surge como un campo innovador dentro de la enfermería. **(2)** La EP representa la transición de una atención estandarizada a un enfoque altamente personalizado. **(3)** La enfermería está estratégicamente posicionada para liderar la traducción de este avance. **(4)** La EP reafirma una atención de enfermería más personalizada, predictiva y participativa. **(5)** La EP puede consolidarse como un pilar en la transformación de la Enfermería Moderna.

La Enfermería de Precisión (EP) surge como un campo innovador dentro de la Enfermería, impulsada por los avances en la Medicina de Precisión (MP) y las ciencias “ómicas”^(1-2)^. La EP representa la transición de la atención estandarizada a un enfoque altamente personalizado basado en datos clínicos, fisiológicos, ambientales, sociales y conductuales^(3)^. Este cambio enfatiza no solo el tratamiento, sino también la promoción de la salud, la prevención de enfermedades y la intervención temprana. Además, la EP se centra en la atención personalizada, que requiere que los enfermeros actúen con precisión y competencia al tratar con pacientes con diferentes necesidades^(2-3)^. Por lo tanto, la EP se basa en la singularidad de cada individuo, teniendo en cuenta su historial de salud, sus preferencias y la mejor evidencia científica disponible, para brindar atención personalizada a lo largo del ciclo vital^(1-3)^. La transición de la genómica estructural a la funcional ha permitido integrar diversas ciencias ómicas, como la proteómica, la epigenómica, la transcriptómica, la farmacogenómica y la metabolómica, a la práctica clínica, y marca el comienzo de una nueva era de atención personalizada^(1,3)^.

La enfermería, gracias a su proximidad al paciente y su experiencia en atención holística, se encuentra estratégicamente posicionada para liderar la traducción de estos avances en intervenciones clínicas eficaces y seguras. La EP no solo redefine la atención personalizada, sino que también amplía el alcance de la práctica basada en la evidencia al incorporar biomarcadores y herramientas tecnológicas para un monitoreo más preciso y eficaz^(1-2)^.

Enfermeros de todo el mundo están ampliado su trabajo para identificar biomarcadores asociados con diferentes afecciones, como estrés crónico en niños y adolescentes, fragilidad en los ancianos, mantenimiento del acceso vascular, prevención de lesiones por presión y deterioro cognitivo^(1-3)^. Otros profesionales de enfermería han explorado la inteligencia artificial para predecir el riesgo de caídas y lesiones por presión, además de investigar biomarcadores en intervenciones no farmacológicas para optimizar los resultados clínicos^(4-5)^. Además, el foco en la prevención, el manejo y el alivio de los síntomas es una de las principales contribuciones en el campo de la EP, dirigida a reducir los síntomas y mejorar la calidad de vida^(5)^.

La incorporación de estas tecnologías a la práctica de enfermería diaria refuerza la capacidad del enfermero para brindar una atención más predictiva, preventiva y participativa^(1)^. A pesar del potencial transformador de la EP, aún existen desafíos. Tales como, que es necesario contar con profesionales capacitados específicamente en ciencias ómicas, integrar datos de múltiples fuentes para personalizar la atención y que haya equidad en el acceso a las nuevas tecnologías^(1-2)^. Es fundamental fortalecer la infraestructura de datos y adoptar modelos de investigación traslacional para garantizar la implementación efectiva de la EP en la práctica clínica^(3)^.

Tradicionalmente, los datos clínicos se obtienen mediante métodos como cuestionarios estandarizados, registros de servicios de salud, historias clínicas electrónicas, informes ómicos, pruebas de imagen y monitoreo fisiológico. Sin embargo, se han incorporado tecnologías emergentes como los dispositivos móviles de salud (*mHealth*), sensores no invasivos como *actigraphy* y sistemas de monitoreo pasivo para proporcionar un análisis más detallado de las características biológicas, conductuales y ambientales de las personas^(1-2)^. Dicha información es fundamental para personalizar las intervenciones, lo que permite que el manejo de los síntomas y el autocuidado sean más eficaces para las personas con mayor probabilidad de responder positivamente a las estrategias terapéuticas^(1-2)^.

También es necesario discutir sobre la equidad y la salud de precisión, considerando que los factores socioeconómicos, culturales y ambientales influyen significativamente en la respuesta al tratamiento^(1,6)^. Otro desafío importante es incorporar la tecnología en la Era de la Precisión, lo que plantea importantes cuestiones éticas e impactos en los sistemas de salud a nivel mundial^(1,6)^.

Formar y capacitar enfermeros en ciencias ómicas y tecnologías disruptivas en las que se basa la atención personalizada es un desafío. Para lograrlo, es fundamental reformular la currícula de las carreras de grado y posgrado, integrando dichos conocimientos y preparando a la fuerza laboral para trabajar en EP^(1,7)^. La educación continua y el desarrollo profesional son esenciales para que los enfermeros identifiquen áreas de aplicación, realicen investigaciones y promuevan la innovación en salud^(7)^. Entre las áreas de mayor desarrollo de la EP se destaca la Oncología, en la que los enfermeros abordan directamente las ciencias ómicas para la prevención, el diagnóstico y el tratamiento del cáncer, al utilizar pruebas genéticas, biomarcadores, farmacogenómica, proteómica y bioinformática^(1,7)^.

Un aspecto fundamental para consolidar la EP es transferir el conocimiento generado mediante la investigación a la práctica clínica, para facilitar la toma de decisiones y brindar una atención personalizada^(1,7)^. A pesar de los desafíos, la investigación traslacional en enfermería puede generar beneficios significativos para los pacientes y sus familias, e impactar positivamente en la calidad de vida y los resultados clínicos.

La EP representa un enfoque innovador que tiene el potencial de transformar la atención médica. Sin embargo, su implementación requiere desarrollar competencias especializadas, incorporar tecnologías emergentes y fortalecer políticas que garanticen la equidad en el acceso a las innovaciones. Al superar estos desafíos, la Enfermería puede consolidar su liderazgo en la promoción de una atención cada vez más personalizada, eficiente y humanizada.

Por lo tanto, la Enfermería de Precisión no es un concepto ajeno a la práctica diaria de los enfermeros. Al contrario, es un enfoque viable y necesario para promover la atención centrada en la persona, basada en evidencia sólida y en la incorporación de nuevas tecnologías. Tras superar desafíos como la capacitación y la infraestructura de datos, la Enfermería de Precisión puede consolidarse como un pilar fundamental en la transformación de la Enfermería moderna.

## Data Availability

Todos los datos generados o analizados durante este estudio están incluidos en este artículo publicado.
